# The Sensorimotor System Can Sculpt Behaviorally Relevant Representations for Motor Learning

**DOI:** 10.1523/ENEURO.0070-16.2016

**Published:** 2016-08-23

**Authors:** David W. Franklin, Alexandra V. Batchelor, Daniel M. Wolpert

**Affiliations:** 1 Computational and Biological Learning Laboratory, Department of Engineering, University of Cambridge, Cambridge CB2 1PZ, United Kingdom; 2 Neuromuscular Diagnostics, Department of Sport and Health Sciences, Technical University of Munich, 80992 Munich, Germany; 3 Department of Physiology, Development and Neuroscience, University of Cambridge, Cambridge CB2 3EG, United Kingdom

**Keywords:** coordinate frame, dynamics, motor learning

## Abstract

The coordinate system in which humans learn novel motor skills is controversial. The representation of sensorimotor skills has been extensively studied by examining generalization after learning perturbations specifically designed to be ambiguous as to their coordinate system. Recent studies have found that learning is not represented in any simple coordinate system and can potentially be accounted for by a mixed representation. Here, instead of probing generalization, which has led to conflicting results, we examine whether novel dynamics can be learned when explicitly and unambiguously presented in particular coordinate systems. Subjects performed center–out reaches to targets in the presence of a force field, while varying the orientation of their hand (i.e., the wrist angle) across trials. Different groups of subjects experienced force fields that were explicitly presented either in Cartesian coordinates (field independent of hand orientation), in object coordinates (field rotated with hand orientation), or in anti-object coordinates (field rotated counter to hand orientation). Subjects learned to represent the dynamics when presented in either Cartesian or object coordinates, learning these as well as an ambiguous force field. However, learning was slower for the object-based dynamics and substantially impaired for the anti-object presentation. Our results show that the motor system is able to tune its representation to at least two natural coordinate systems but is impaired when the representation of the task does not correspond to a behaviorally relevant coordinate system. Our results show that the motor system can sculpt its representation through experience to match those of natural tasks.

## Significance Statement

The nature of the coordinate system in which humans learn motor skills has been highly controversial. Despite extensive experimental work, the results are highly conflicting (e.g., intrinsic joint-based vs Cartesian vs mixed coordinates). In our study, we show that the motor system is able to sculpt and tune its representation to at least two natural coordinate systems. Importantly, we also show that learning is impaired for a task of equal complexity that does not correspond to a naturalistic coordinate system. Our results suggest that the previously conflicting findings arise primarily from the use of novel skills that are ambiguous as to their coordinates, thereby making the experimental results highly sensitive to small differences in the experimental design.

## Introduction

The search for the coordinate system of motor control has been one of the holy grails of sensorimotor neuroscience at both the neural level (Kakei et al., 1999; Hatsopoulos, 2005; Aflalo and Graziano, 2007; Scott, 2008; Kalaska, 2009; Graziano, 2011; Churchland et al., 2012) and the behavioral level (Shadmehr and Mussa-Ivaldi, 1994; Malfait et al., 2005; Brayanov et al., 2012; Berniker et al., 2014), often with conflicting interpretations. Many studies have tried to identify the coordinate system in which motor skills are encoded by examining the generalization of learning. Although initial experiments suggested that the representation of novel dynamics occurs in simple intrinsic coordinates (i.e., in terms of joint torques and joint velocities; Shadmehr and Mussa-Ivaldi, 1994; Malfait et al., 2002, 2005), recent studies have questioned these results (Haswell et al., 2009; Brayanov et al., 2012; Berniker et al., 2014; Parmar et al., 2015). For example, Berniker et al. (2014) examined whether generalization could be accounted for by the sensorimotor system representing dynamics in intrinsic joint, extrinsic Cartesian, or object-based representations. They found that no single coordinate frame could explain the generalization of dynamics and that the representation was best accounted for by a mixture of these coordinate systems (Parmar et al., 2015). Such a mixed representation is in accord with studies of visuomotor learning, which has been shown to result from a mixed gain field encoding of intrinsic and extrinsic coordinates (Brayanov et al., 2012). Critically, all of these previous studies have used perturbations that are designed to be ambiguous as to the representation of the task (e.g., a skew-viscous curl force field in a small workspace or a force field for a single reach direction), and, therefore, these perturbations could in theory have multiple different representations. Therefore, the conflicting results may well reflect the participant’s personal biases as well as small differences in the experimental conditions and perturbations.

Here we take a novel approach and, instead of probing generalization, we examine how well novel dynamics can be learned when the dynamics are explicitly and unambiguously presented in particular natural and unnatural coordinate systems. This allows us to test whether the motor system can learn to represent dynamics in specific different coordinate systems. That is, previous work has suggested that motor adaptation occurs in a mixture of coordinate frames (Brayanov et al., 2012; Berniker et al., 2014; Parmar et al., 2015). In particular, in the study by Berniker et al. (2014) ambiguous dynamics were presented to subjects to determine how such learning generalizes, and the results were consistent with subjects using a mixture of coordinate systems to learn the dynamics. The authors suggested that a specific skill could be learned by an appropriate combination of the coordinate system for that skill. Here we compare, for the first time, the learning of an ambiguous field and several nonambiguous fields. This allowed us to examine directly the extent to which the proposed mixed representation could be shaped to learn specific fields. By providing the dynamics unambiguously to the subjects, we determine whether the subjects can learn to use a specific coordinate frame, or, in other words, sculpt the particular coordinate system in which the task is learned.

Subjects performed center–out reaching movements to the same targets, but with three different hand orientations (neutral, flexed, and extended). The force field that we generated at the hand could be independent of these hand orientations, and therefore the experience was of an unambiguous Cartesian force field. Alternatively, we could vary the force field in real time with the hand orientation so that the force field either rotated with the hand, as if the force field was generated by a hand-held tool (natural object-based coordinates), or rotated counter to the hand (unnatural anti-object coordinates). Our results clearly show that the motor system can represent dynamics equally well in different natural coordinate systems (Cartesian and object), but not in an arbitrary coordinate system (anti-object), even when matched for complexity. This suggests that the motor system can sculpt different natural coordinate systems that are appropriate to task demands.

## Materials and Methods

Forty naive subjects (17 male and 23 female) participated in the experiment (mean age, 25.9 ± 5.8 years), with 10 subjects assigned randomly to each of four groups. All subjects were right handed, according to the Edinburgh handedness inventory (Oldfield, 1971). Subjects gave informed consent, and the experiments were approved by the local ethics committee.

### Experimental setup

Subjects were seated in a chair with their shoulders restrained by a harness. They made reaching movements with their right arm in the horizontal plane at ∼10 cm below their shoulder level, while the forearm was supported by an air sled. Subjects grasped the vertical handle of the robotic manipulandum (vBOT), which was used to generate the environmental dynamics (Howard et al., 2009). The handle was free to rotate around its vertical axis, and its position and orientation in extrinsic space was measured using optical encoders on the handle (58SA, IED) and motors. A fixed hand support was attached to the handle to ensure that the handle could only be grasped in a single way so that we could measure and control the hand orientation. End point forces at the handle were measured using an ATI Nano 25 six-axis force–torque transducer (ATI Industrial Automation) mounted below the handle. The handle position and orientation, and the force data were sampled at 1 kHz. Visual feedback was provided using a computer monitor mounted above the vBOT and projected veridically to the subject via a mirror.

**Figure 1.**
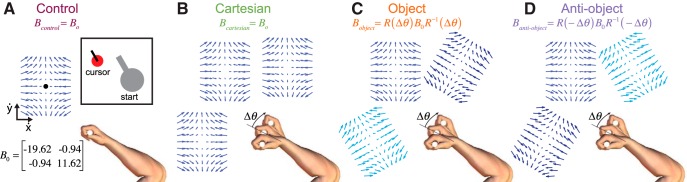
Experimental design and learning. ***A–D***, Hand orientations and associated force fields, as force vectors experienced by the hand (zero velocity represented by the black circle) for the four groups of subjects. Note that the three vector fields are displayed separately, for clarity, but act over the same workspace. Similarly, the actual end point location of the hand for the three hand orientations is matched across the three conditions, but is separated here for clarity. Start location, targets, and cursor were displayed as a circle, showing position with a line indicating the orientation (inset).

The hand cursor was displayed as a circular disk (0.5 cm radius) with a small bar emanating from the disk displaying the orientation of the handle (i.e., hand). Similarly, the start location and target were also displayed as circular disks (1.0 cm radius), each with a bar indicating the required hand orientation at the start and end of a movement (Fig. 1*A*, inset). On all trials, the start and target orientation were identical but could vary between trials. Prior to movement, the hand was required to be within the start location (positional tolerance, 0.5 cm) and with orientation bars aligned (orientation tolerance 5°) for 750 ms. A tone sounded to initiate the trial. Movements were made from the central start location to one of six possible targets equally spaced around a circle with a 10 cm radius. A movement ended once the hand was at the target (positional tolerance, 0.8 cm; orientation tolerance, 12.5°). Feedback was given as to whether the movement was too slow or fast, and if the peak speed was 50 ± 8 cm/s, a reward counter increased. The robot passively moved the hand back to the start position. Subjects took short breaks every 100 trials.

### Paradigm overview

For the control group, all movements were made with the same hand orientation, whereas, for the other three groups, each movement was performed with three different hand orientations. The three hand orientations were a neutral position and ±30°. The neutral orientation was with the hand slightly flexed, so that all postures could be comfortably reached. Initially, subjects performed 36 familiarization trials in a null field. Importantly, the end point location for movements in all three hand orientations were matched in Cartesian space. This means that for each set of hand orientations, the shoulder and elbow joint motions were slightly different.

**Figure 2.**
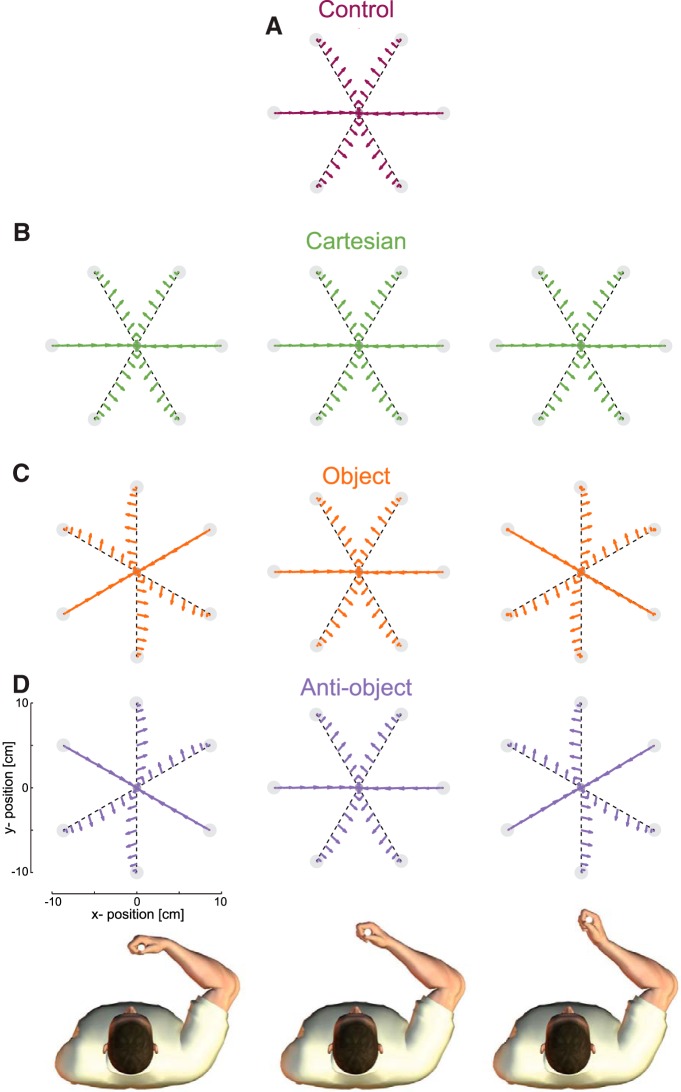
Force field conditions and movements across the experimental design. The wrist configuration (flexed, neutral, and extended) is shown for the three columns at the bottom of the figure. ***A***, The control group always performed movements in six directions with the neutral wrist configuration. ***B***, The Cartesian group performed reaching six reaching movements with the same Cartesian direction and forces for all three wrist orientations. ***C***, The object group. The six reaching directions were rotated for each wrist orientation such that the forces experienced were identical to the Cartesian condition (but rotated by 30°). ***D***, For the anti-object group, the force field and the reaching directions rotated in the opposite direction to the wrist. However, the forces experienced still matched those of the other groups. Importantly, the force fields and reaching directions on the middle wrist configuration were identical across all four groups.

The experiment was performed in two stages running consecutively, as follows: a pre-exposure phase (240 trials) was followed by the exposure phase (1320 trials). The pre-exposure phase was performed in a null field, while the exposure phase had a velocity-dependent force field. The exact nature of this force field varied between the experimental groups. Throughout the pre-exposure and exposure trials, blocks of trials were performed that included field trials (to all six targets) and channel trials (to four of the six targets). Channel trials were only used for the four targets in which significant lateral forces were applied by the force field. In the other two directions, a resistive force was produced, making channel trials uninformative (Fig. 2*A*). On a channel trial, the movement was confined to a simulated mechanical channel with a spring constant of 8000 N/m and damping of 2 Ns/m (Scheidt et al., 2000; Milner and Franklin, 2005).

In the pre-exposure phase, each block (48 trials) consisted of 36 field trials (2 to each of the six targets with each of the three hand orientations) and 12 channel trials (1 to each of the four targets with each of the three hand orientations). In the exposure phase, each block (66 trials) consisted of 54 field trials (3 to each of the six targets with each of the three hand orientations) and 12 channel trials (1 to each of the four targets with each of the three hand orientations). Within a block, the order of the trials was pseudo-randomized while ensuring that two channel trials never occurred consecutively. A different pseudo-random sequence of trials was used for each subject.

### Control group

For the control group, only the neutral hand orientation was used, and these subjects performed the same total number of reaching movements as all other groups. This group performed reaching movements to the six targets, and the force field dynamics were given by the following:

$$\begin{array}{l} F=B\cdot \dot{x}\\ B=\left[\begin{array}{cc} -19.62 & -0.94\\ -0.94 & 11.62 \end{array}\right] \end{array}$$

where F is the vector of *x-* and *y-*forces applied to the hand in *N*, and $\dot{x}$ is the vector of hand velocities in meters per second (Fig. 1, vector field shown as a function of hand velocity with stationary shown by the central dot). This skew-viscous force field resists movements in some direction, assists in others, and acts at an angle to the movement direction for yet other movement directions (Fig. 2*A*). Critically, unlike a viscous curl field, the skew-viscous field has a clear orientation (e.g., the axis of movement that is purely resistive). As this group of subjects performed the movements with only one hand orientation (the neutral one), in common with previous studies, the coordinate system of the dynamics is ambiguous (Shadmehr and Mussa-Ivaldi, 1994; Berniker et al., 2014).

### Cartesian group

The Cartesian group of subjects performed reaching movements to the six targets with three different hand orientations (18 total conditions). The six targets were equally spaced at 60°, and the location of the targets was maintained for all hand orientations. The forces experienced differed across the six movements but, critically, did not vary with the hand orientation, and therefore the field is unambiguously consistent with forces represented in a Cartesian frame (Figs. 1*B*, 2*B* ). Importantly, the end point location of the hand for the three hand orientation conditions is matched, meaning that the elbow and shoulder angles are slightly different for these conditions. However, the wrist angle (intrinsic coordinates) is very different for the three orientations, and the field is independent of this angle, meaning that the Cartesian representation is distinguishable from an intrinsic or joint-based representation.

### Object group

The object group of subjects also performed reaching movements to six targets with the three hand orientations. The force field was implemented as follows:

$$F=RBR^{-1}\dot{x}$$

where *R* is the rotation matrix that corresponds to the hand orientation and thereby rotates the force field by the same angle as the instantaneous hand orientation. Although subjects were required to maintain the orientation of the hand at the start and end of the moment, during each movement small variations from this orientation were tracked and used to update the force field on-line within a movement. In this manner, the force field rotates with the orientation of the hand, identical to the way in which the dynamics of a grasped object would rotate as the hand orientation changed (Fig. 1*C*). We chose to rotate the targets with the required hand orientation (±30°) so as to ensure that the forces experienced in this condition were identical to the Cartesian condition with the exception that the association of the forces to the movement varied with the hand orientations (Fig. 2*C*). This ensures the mathematical complexity to be constant across groups. The forces matched those of the Cartesian group for the same target directions for the neutral hand position. Therefore, for this group the dynamics are unambiguously consistent with forces that rotate with the hand orientation.

### Anti-object group

The anti-object group of subjects performed reaching movements similar to those performed by the object group, with the exception that the force field rotated in the opposite direction to the rotation of the hand (Fig. 1*D*). This produced force fields that were identical to those of the object group, but that were associated with different hand orientations (Fig. 2*D*). Therefore, the mathematical complexity of this field is the same as that for the object group, but rotates in the opposite direction to the way in which a hand-held tool would behave. Again, the targets rotated with the desired hand orientation, and the forces matched those of the Cartesian group for the same target directions in the neutral hand position. Note that, as the targets are spaced at 60° intervals, ±30° rotations of the targets are identical.

### Analysis

Analysis of the experimental data was performed using Matlab R2015a. Individual trials were aligned on movement onset. For each nonchannel trial, the maximum perpendicular error (MPE) was calculated and used as a measure of the kinematic error. The MPE is the signed maximum perpendicular distance between the hand trajectory and the straight line between the start and end targets. The sign of the measurements for different movement directions and hand orientations was adjusted so that positive numbers reflected errors due to the perturbing force field. The end point forces were examined on the channel trials to further measure the amount of adaptation. The force produced by subjects into the wall of the simulated channel was integrated across the movement. To evaluate the degree of compensation (Smith et al., 2006), the measured force was divided by the amount of force that would be required for perfect compensation in the force field (calculated as the force field strength multiplied by the actual velocity on each trial). The values of the percentage force compensation throughout the experiment are based on the compensation required in the force field. Therefore, values in the null force field before learning (pre-exposure phase) should be close to zero.

We performed hypothesis-based planned comparisons and report uncorrected *p* values to determine statistical significance (*p* < 0.05). ANOVAs were examined in SPSS (version 21) using the general linear model. Comparisons were made with main effects of epoch (pre-exposure, early exposure, or late exposure), force field condition (four levels), movement direction (six levels), and hand orientation (three levels), when appropriate. For the object and anti-object groups, the targets rotated with the hand orientation and for the ANOVA we matched up the six directions across orientations so that they had the same end point forces (for a straight line movement). Early and late exposures correspond to the first and last 18 trials in the force field. The final level of adaptation was examined across the final 330 trials (with MPE and force compensation calculated from 270 and 60 trials, respectively). If a significant main effect was found, Tukey’s HSD *post hoc* test was used to examine differences.

The mean MPE and force compensation across subjects during the exposure phase of the experiment were fit with the following exponential functions:

$$y=\alpha \cdot e^{-\lambda \cdot k}+C$$

where C is the asymptote of final adaptation, λ is the time constant or rate of adaptation, k is the trial number, and α is the magnitude of adaptation. The exponential function was fit using the Matlab function nlinfit, and 95% confidence intervals (CIs) of the function and the parameters were determined using nlpredci, and nlparci, respectively. To examine the statistical differences between our parameter estimates, we performed block bootstrapping in which we left out each possible set of three participants from each group and fitted the remaining seven. To test whether each parameter varied between the four groups, we generated all possible differences in each parameter from our bootstrap to generate a new bootstrap sample (120 × 120 samples). The six comparisons were then examined at a Bonferroni-adjusted statistical level (*p* < 0.0083).

## Results

**Figure 3.**
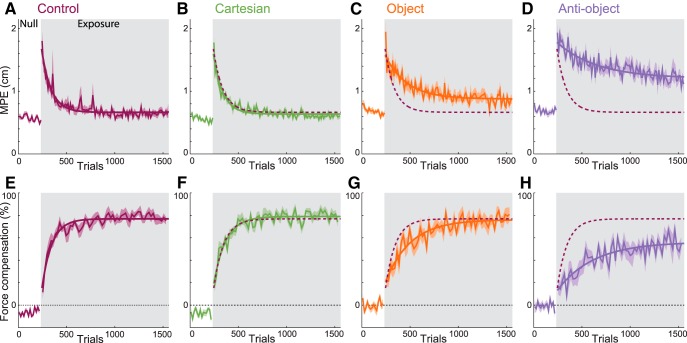
Adaptation to the force fields. ***A–D***, Maximum perpendicular error. ***E–H***, Force compensation percentage for the four groups (mean and SE across subjects) with exponential fits. Blocks of 10 nonchannel trials were used for the MPE, while blocks of four channel trials were used for the force compensation. The gray shaded region indicates the exposure period. The dashed curves are the exponential fit to the control group for comparison.

Subjects performed center–out reaching movements to six targets, and were required to match both the position and orientation at the start and end of each reach. For the control group, all movements were made with the same neutral hand orientation (Fig. 1*A*), whereas for the other three groups, each movement was performed with one of three different hand orientations (neutral position and ±30°; Fig. 1*B*). For all groups, movements in the null field were approximately straight (Fig. 3*A–D*, first 240 trials).

For the Cartesian group, the force depended on hand velocity but, critically, did not vary with the hand orientation, and, therefore, the field is unambiguously consistent with forces represented in a Cartesian frame (Fig. 1*B*). For two of the groups, the entire force depended on the hand orientation, either rotating with the hand (Fig. 1*C*, object-group), consistent with the dynamics of a hand-held object, or counter to the hand rotation (Fig. 1*D*, anti-object).

**Figure 4.**
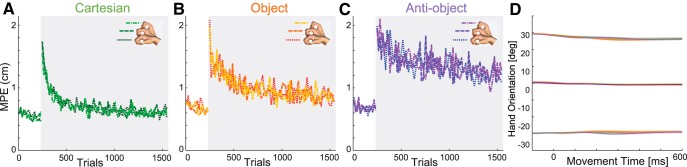
Learning and kinematics as a function of hand orientation. ***A–C***, Learning curves separated by hand orientation for three groups. ***D***, Hand orientation (mean ± SE averaged as in Fig. 1) for the four groups as a function of time (colors are as in Fig. 1).

On the introduction of a force field in the exposure phase, large increases in MPE were observed (Fig. 3*A–D*, gray region) that were not significantly different across the four groups (*F*_(3,660)_ = 0.864; *p* = 0.46; initial exposure). Across trials, the MPE was reduced, and we compared the final levels of adaptation (last 270 trials) using an ANOVA with factors of group, movement direction, and hand orientation. Only the main effect of group was significant (*F*_(3,107,400_ = 620.01; *p* < 0.001), with no significant effect of wrist angle (*F*_(2,21,540)_ = 0.86; *p* = 0.42), movement direction (*F*_(6,21,540)_ = 0.62; *p* = 0.69), or interactions (all *p* > 0.09). *Post hoc* tests indicated that the final error for the anti-object group was significantly greater than all others (all *p* < 0.001). The object group had a significantly larger final error than both the Cartesian and control groups (*p* < 0.001). The Cartesian group had a slightly smaller error than the control groups (*p* = 0.016). Importantly, the learning within each group was similar for each of the hand orientations (Fig. 4*A–C*), and subjects were able to control the hand orientation throughout the movements (Fig. 4*D*).

To examine the feedforward component of learning, independent of any nonspecific changes in arm impedance (Franklin et al., 2003, 2007), we examined the lateral force produced on occasional channel trials (Scheidt et al., 2000; Milner and Franklin, 2005) and calculated the percentage compensation for the force field (Fig. 3*E–H*). In the pre-exposure phase, the subjects generated minimal force into the channel wall (Fig. 3*E–H*, first 240 trials). At the end of the exposure phase, there was a significant difference in the final force compensation values across the groups (*F*_(3,2360)_ = 72.66; *p* < 0.001, last 60 channel trials, corresponding to the same interval as the 270 trials examined for MPE). *Post hoc* tests showed that the compensation for the anti-object group was significantly less than all others (all *p* < 0.001). There were no significant differences between the Cartesian and object groups (*p* = 0.1) or between these two and the control group (*p* = 0.82 and *p* = 0.47 respectively).

**Figure 5.**
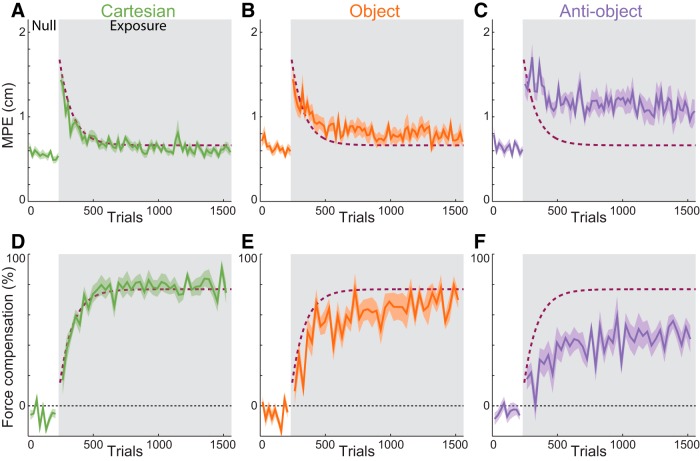
Learning and force compensation for the neutral wrist orientation where the force field is identical across all four groups. ***A–C***, The maximum perpendicular error (mean ± SE across subjects). The dotted line shows the exponential fit for the control group for comparison. ***D***, ***E***, Force compensation.

Importantly, our experimental design meant that all groups experienced the same movements and forces for the neutral wrist orientation (Fig. 2). As such, we can directly compare the adaptation that occurred only in this single wrist posture, thereby controlling for both the movement and field experienced (Fig. 5). The results demonstrate that the same findings across all wrist postures (Fig. 3) are also present in this single wrist posture condition.

**Figure 6.**
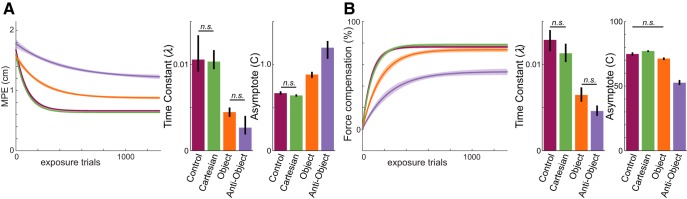
Time constants and asymptotes of learning across conditions. ***A***, Left, The mean (±95% CI) of the exponential fits to the group-averaged MPE for the exposure phase. Right, The asymptote and time constant (±95% CI) of the exponential fits (all comparisons were significant at the *p* < 0.0083 level, except for those shown with the flat bar and label *n.s.* indicating that the comparison was not significant after Bonferroni correction). ***B***, Fits and parameters for the force compensation data as in ***A***.

To compare the rate and final level of learning across all groups, we fit exponential curves to both the kinematic error and force compensation during exposure trials (Fig. 3, smooth curves show the fits and dashed curve is fits to the control group for comparison). Figure 6 shows the fits for the four groups with 95% CIs. We used bootstrap analysis (see Materials and Methods) to compare the time constants and asymptotes across the groups. For the kinematic error (Fig. 6*A*), the time constants for the control and Cartesian groups were not significantly different from one another (*p* = 0.38) and were significantly shorter than those for either the object or anti-object groups (all *p* < 0.00001). The object group time constant was not different from that for the anti-object group (*p* = 0.09). For the final maximum perpendicular error level (asymptote), the lowest asymptote was seen in the Cartesian and control groups (not significantly different; *p* = 0.16), while the object group had a significantly higher asymptote (all *p* < 0.00001). Again, the largest asymptote was found for the anti-object group (all *p* < 0.004).

A similar pattern was found across the parameters determined from fits to the force compensation data (Fig. 6*B*). For the time constants, there was again no difference between the Cartesian and control groups (*p* = 0.18); however, both groups were significantly faster than the object or anti-object groups (all *p* < 0.00001). Again, no significant difference was found between the object and anti-object groups after multiple comparison correction (*p* = 0.03). There were significant differences in the level of final adaptation (asymptote). There was no difference between the control group and the Cartesian group (*p* = 0.05) or the object group (*p* = 0.01) after correction; however, there were significant differences between the Cartesian and object groups (*p* < 0.0001). The anti-object group had significantly lower final adaptation than all three groups (all *p* < 0.00001).

Interestingly, there were strong differences between the results based on the maximum perpendicular error and those based on predictive force compensation. While these two measures of learning normally give similar results, the kinematic error can be reduced independent of predictive force compensation by increasing the stiffness of the limb by cocontraction or increased feedback gains during reaching (Burdet et al., 2001; Franklin et al., 2003, 2012). In our study, while the force compensation results suggested that the object group learned as well as the Cartesian and control groups, the final level of kinematic error measure was still higher. This could indicate that the cocontraction was lower in this object group, but other biomechanical factors related to limb geometry (Burdet et al., 2013) could also have played a role. In either case, the difference in final adaptation for the object group was only on the order of 2 mm.

## Discussion

Although early studies have suggested that force field adaptation is learned in intrinsic coordinates (Shadmehr and Mussa-Ivaldi, 1994; Malfait et al., 2002, 2005), our results clearly show that a force field explicitly presented in Cartesian coordinates is learned on a very similar timescale and with levels of learning similar to those of an ambiguous force field (Fig. 3*E–H*). An ambiguous force field would be learned in the preferred coordinate system (e.g., intrinsic) if such a preferred coordinate system actually exists. In other words, if the dynamic learning is represented in intrinsic coordinates, then we would have expected the Cartesian condition to be more difficult to learn and have a higher final error than the ambiguous field. A similar argument can be made for the field presented in object-based coordinates, where the final force compensation level was not significantly different from that of the Cartesian or control group. Therefore, our results suggest that both the Cartesian group and the object group learned to represent the dynamics in the coordinate system in which they were presented, demonstrating a flexibility to sculpt the coordinate system when coding dynamics.

We consider the object-based force field as a singular dynamic experienced in three different hand orientations. However, another possibility is that the sensorimotor system represents this as three separate force fields learned independently for each hand orientation. This would place our findings in agreement with those demonstrating that hand orientation (Gandolfo et al., 1996; Yeo et al., 2015) or similar differences in the limb state (Hwang et al., 2003; Howard et al., 2013) can be used to reduce interference and learn opposing force fields, although extending them to three states. However, if this was the case for the object-based force field, then the anti-object-based force field should have been equally easy to learn as the three separate context-dependent fields. Instead, the demonstration that the anti-object force field is not learned equally well shows a limitation in the ability of small changes in hand orientation to allow for independent learning of different force fields over a similar state space. Our results indicate that force field presentations that do not occur naturally are not as quickly or well learned compared with those that can be represented in a more natural coordinate frame.

We propose that ambiguous dynamics are represented in a mixture of coordinate representations (Haswell et al., 2009; Brayanov et al., 2012; Berniker et al., 2014; Parmar et al., 2015), such as Cartesian, intrinsic, and object based, with the weighting of this mixture being driven by the likelihood of each particular representation. Such a mixture would provide a rapid adaptation to the ambiguous force field with the mixing components then appropriately reweighted if the force field became unambiguous (as in the Cartesian and object groups). However, such a mixed representation would struggle to learn representations that could not be achieved through mixing (anti-object).

Our findings shed light onto why so many conflicting results about the nature of coordinate representation were found, such as evidence for both intrinsic (Shadmehr and Mussa-Ivaldi, 1994; Shadmehr and Moussavi, 2000; Malfait et al., 2002, 2005) and extrinsic (Krakauer et al., 2000; Criscimagna-Hemminger et al., 2003; Burgess et al., 2007) generalization. Instead, our work further supports the idea that dynamics can be represented in a mixture of coordinate systems (Haswell et al., 2009; Brayanov et al., 2012; Berniker et al., 2014), of which intrinsic, extrinsic, and object coordinate systems are three of the (possibly many) representations available. Our results jibe with recent neurophysiological studies showing that many neurons show complex mixed selectivity in motor tasks (Churchland et al., 2012) and more cognitive tasks (Mante et al., 2013; Rigotti et al., 2013). Although the results of the anti-object group clearly preclude the possibility that any arbitrary mapping can be learned on a short time scale (∼2 h), a critical question is whether these coordinate representations are fixed a priori or whether they can be learned with sufficient experience.

## Synthesis

The decision was a result of the Reviewing Editor Trevor Drew and the peer reviewers coming together and discussing their recommendations until a consensus was reached. A fact-based synthesis statement explaining their decision and outlining what is needed to prepare a revision is listed below. The following reviewers agreed to reveal their identity: John Kalaska, Denise Henriques

The authors studied the learning rates of 4 groups of subjects that performed reaching movements in different ranges of external skew-viscous force fields (FFs) and hand/wrist configurations. They found that the "Control" group that made reaching movements in only one FF with one arm configuration learned very rapidly. A second group ("Cartesian") that performed reaching movements in the same FF as the control subjects (i.e., fixed in Cartesian space) learned almost as well as the control group despite experiencing the FF while holding their hand in one of three wrist postures. The third group ("Object") also learned to compensate for FFs that rotated with the wrist posture, as if the FFs were coupled to the spatial orientation of a fictive object held in their hand, except that the learning rate was slower than for the first two groups. The fourth group ("Anti-object") encountered FFs that rotated in the direction opposite to the posture of the hand and showed only slow, partial compensation for the fields.

These data are interesting and are a useful contribution to the field. However, I have to question whether they are as novel and insightful as the authors claim in the manuscript.

First, the authors should provide a clearer explanation of how these new results provide a better insight into the coordinate framework(s) for motor learning than their earlier study that used generalization to try to assess frameworks for learning (Berniker et al 2014). I think I know, but they do not express it explicitly enough. They should also provide a clearer explanation of what they mean by the title of the manuscript. What does "sculpt behaviorally relevant representations for motor learning" mean and how does that advance our understanding of motor learning? They use the word "sculpting" a number of other times in the text. How does that differ from the conclusion in Berniker et al 2014 that subjects may use a task-specific mixture of frameworks to learn a particular motor skill? Isn't that also "sculpting a behaviorally relevant representation", but just expressed differently?

The authors frequently refer to the FF for the Control subjects as "ambiguous" because they only sample it in one arm/hand configuration. However, in the way that they present the learning problem in the manuscript (e.g. ln 34-36, 173-174), one might actually expect that this ambiguity could put them at a disadvantage with respect to other subjects, especially the Cartesian group who experience it in 3 different hand configurations and can then better define its spatially-constant nature. Why then is the performance of the 2 groups essentially equivalent? Alternatively, maybe that result is not entirely surprising? It is not clear from the description of the task, but when the Cartesian subjects hold their hand in 3 different postures, do they also make reaching movements along slightly different compensatory spatial trajectories and correspondingly different shoulder/elbow joint rotations and torques, or do they in fact follow the same trajectories in shoulder/elbow joint velocity/torque space and the hand configurations have relatively little impact other than requiring small adjustments of forearm/wrist muscle activity to stabilize the wrist in the different postures? If it is the latter case, then the similar performance could be expected because the wrist postures are largely irrelevant to nature of the learning task. As a result, I don't understand why they state that the Cartesian field should be more difficult to learn if learning is in intrinsic coordinates because it is not evident how the change in hand posture impacts on the control of the shoulder and elbow joints (ln 398-401), which might actually be relatively constant across hand postures. This needs to be clarified.

The authors argue that the "Object" subjects learn the task because it is fixed in "object" space and so could be learnable. However, one could also consider this to be yet another demonstration that different hand or arm geometries provide sufficient "contextual" cues to permit simultaneous learning of multiple different and even anti-correlated FFs. The first such study that I can think of is Gandolfo et al 1996, but there have been several others in the ensuing years that the authors undoubtedly know better than me. This convergence of findings should be acknowledged in the manuscript. As for the slower learning rate, this may have to do with the fact that the FFs are in object-centered coordinates rather than Cartesian coordinates and so might be slightly more difficult to learn. Once again, however, if the reach trajectories are relatively constant in shoulder/elbow joint/torque space and most of the learning occurs in that coordinate framework, this means that the subjects only practice in each of the 3 FFs one third as often as the Control and Cartesian subjects, so slower learning should be expected. This point is not raised.

Finally, it is not too surprising that the Anti-object subjects showed the poorest learning because it is unlikely that any of themwould have ever encountered anything like these FFs that appear to contradict the usual laws of physics. On the other hand, the difference in learning between the Object and Anti-object groups demonstrates the limits of hand posture as a context cue to learn different FFs. This point should also be noted.

The trial conditions were varied in a pseudo-random fashion within task blocks. However, did they also vary randomly across subjects or did they present the same trial sequence to all subjects?

The first 2 pages of the Results section (ln 271-309) do not present a single result or data point. It is a summary of the task design that was already described in the Methods section. I acknowledge that it is very well written and clear, but it is not Results. Please incorporate this text into the Methods section.

Similarly, Fig 1A-D and Fig 2 are methodological in nature, while Fig 1E-L present results that fit more naturally with the data in Fig 3. Please restructure those figures more logically.
